# Real-time intraoperative motion-following robotic assistance improves efficiency and accuracy in total knee arthroplasty: a retrospective comparative study

**DOI:** 10.1186/s42836-026-00384-9

**Published:** 2026-04-14

**Authors:** Zihan Li, Zhuwen Xu, Dajiang Li, Hui Shao, Huiwu Li, Keyu Kong, Zanjing Zhai

**Affiliations:** 1 MicroPort NaviBot (Suzhou) Co., Ltd., Suzhou, 215000 People’s Republic of China; 2 Shanghai MicroPort Medbot (Group) Co., Ltd., Shanghai, 201203 People’s Republic of China; 3 Shanghai Key Laboratory of Orthopaedic Implants, Department of Orthopaedic Surgery, Shanghai Ninth People’s Hospital, Shanghai Jiao Tong University School of Medicine, 639 Zhizaoju Road, Shanghai, 200011 People’s Republic of China; 4 Shigatse People’s Hospital, Shigatse, Tibet, 857000 People’s Republic of China

**Keywords:** Robotic-assisted total knee arthroplasty, Motion-following control, Surgical efficiency, Osteotomy accuracy, Dynamic tracking

## Abstract

**Background:**

Conventional robotic-assisted total knee arthroplasty (RA-TKA) relies on rigid limb fixation to suppress intra-operative motion, adding complexity and potential inefficiency. A novel motion-following control system dynamically compensates for limb movement, allowing real-time adjustment of the tool-bone relationship without immobilization. This study evaluated whether motion-following improves efficiency and osteotomy accuracy while preserving alignment and early function.

**Methods:**

Sixty consecutive primary RA-TKA cases performed with the SkyWalker robotic platform (MicroPort, Shanghai, China) between September 2022 and August 2024 were retrospectively reviewed. Thirty procedures used conventional rigid fixation (control group) and thirty employed motion-following tracking (motion-Following group). Primary endpoints were operative time and resection thickness error, measured intraoperatively with a caliper. Secondary outcomes included coronal alignment assessed by HKA (hip-knee-ankle angle), CFCA (coronal femoral component angle), and CTCA (coronal tibial component angle), as well as functional recovery assessed by WOMAC (Western Ontario and McMaster Universities Osteoarthritis Index) at 6 months. Values are expressed as mean ± standard deviation unless otherwise specified.

**Results:**

Mean operative time was shorter with motion-following (118.8 ± 9.3 min) than with conventional fixation (133.9 ± 11.9 min; *p* < 0.001). Mean resection-thickness error was lower with motion-following (0.53 mm vs 0.82 mm), with 93.9% versus 68.3% of cuts within ≤ 1 mm. At the plane level, motion-following achieved smaller errors on all six surfaces, with four planes: DF-M (distal femur medial), distal femur lateral (DF-L), posterior femur medial (PF-M), and tibial plateau lateral (TP-L) reaching statistical significance (*p* < 0.05). Post-operative coronal alignment closely reproduced the pre-operative plan in both groups, with mean deviations of approximately 1° across all parameters and no statistically significant between-group differences. WOMAC scores improved substantially in both groups, with no significant between-group difference (ΔWOMAC 32.8 ± 8.5 vs 30.1 ± 7.9; *p* = 0.21).

**Conclusions:**

Motion-following robotic control streamlines TKA by eliminating rigid fixation, improving workflow efficiency, and slightly enhancing osteotomy precision without compromising alignment or recovery. This dynamic, real-time tracking approach refines execution of the surgical plan and may represent a meaningful evolution toward more efficient, surgeon-friendly robotic arthroplasty.

**Supplementary Information:**

The online version contains supplementary material available at 10.1186/s42836-026-00384-9.

## Introduction

Robot-assisted total knee arthroplasty (RA-TKA), supported by three-dimensional pre-operative planning and high-precision bone resection, is increasingly regarded as a key direction for the future development of knee arthroplasty. It provides consistent advantages in implant positioning, osteotomy accuracy [1, 2], and soft-tissue balancing [3]. Evidence indicates that robotic assistance can improve post-operative pain control, accelerate functional recovery, and reduce length of stay [4]. Longer-term follow-up studies further suggest lower reintervention and revision risks, along with potential cost-effectiveness [5]. RA-TKA also demonstrates superior accuracy in preserving multiplanar knee anatomy and physiological limb alignment [6], without increasing early post-operative complications [7].

Nevertheless, several limitations of this technology remain, among which prolonged operative time and increased procedural complexity are prominent concerns [8]. In most RA-TKA workflows, a long-standing step is the use of a leg holder to rigidly immobilize the operative limb during bone resection [9], aiming to prevent limb motion that could decouple the cutting guide from the intended plane. This requirement is closely related to navigation-based tracking architectures used in many robotic systems, in which bone-mounted reference arrays are monitored relative to a fixed optical camera coordinate system during bone cutting [10]. In practice, however, limb fixation entails time-consuming setup and handling tasks—including mounting, adjustment, and repeated loosening and re-securing for exposure, irrigation, or trialing—which further increase operative time and complexity. Moreover, intra-operative sawblade vibration, soft-tissue traction, changes in knee flexion, or simple positional adjustments may induce subtle micro-motions. Even minor unrecognized shifts can generate registration errors and necessitate intra-operative repositioning, thereby disrupting procedural flow [11]. Because the robot end-effector is typically not independently tracked within the navigation coordinate system, maintaining a stable spatial transformation between the robot base and the navigation system depends on fixed reference arrays and a stationary optical camera. These frictions are amplified in obese patients, constrained operative environments, or when frequent changes in joint positioning are required [12]. If such micro-motions are not detected and corrected promptly, geometric error can propagate into the cut surface [13]. Prolonged operative time has been associated with an increased risk of post-operative complications, including surgical site infection [14]. Thus, enhancing procedural fluidity and efficiency and reducing operative time remain key priorities for future development.

Real-time motion-following (“follow-motion”) control offers a different paradigm that replaces the traditional “fix-and-cut” approach with a dynamic “sense–compute–compensate” control framework [15]. In a motion-following architecture, the robot continuously tracks both the bone and the end-effector with high-frequency sensors and updates the tool pose whenever the limb moves, maintaining a constant relationship between the instrument and the planned cut surface [16]. By tracking both the patient bone and the robot end-effector, this strategy reduces dependence on coordinate-frame calibration between the robot and the navigation system and improves robustness to minor intra-operative displacement of the optical camera. Rather than preventing motion, the system embraces it—synchronizing the tool to the bone and theoretically obviating bulky limb fixation while interrupting the typical “micro-motion → geometric error” cascade [17]. To enable rapid compensation of intra-operative bone displacement, the motion-following system used in this study incorporates a lightweight surgical robotic arm and a high-frequency control architecture. Validation testing performed during development of the motion-following control system demonstrated sub-millimeter tracking accuracy and stable real-time compensation performance under controlled perturbation conditions, suggesting that plan fidelity can be maintained without rigid immobilization. If these properties translate clinically, motion-following control could simplify intra-operative handling, shorten skin-to-skin surgery time and anesthesia time, and further tighten the distribution of resection angle and depth errors, with the potential to support earlier functional recovery by reducing soft-tissue manipulation and workflow interruptions.

To address these questions, we performed a retrospective controlled comparison of RA-TKA performed without versus with motion-following enabled on the same robotic platform. We posited three a priori hypotheses: (i) activating motion-following would eliminate the need for a leg holder by maintaining the planned tool–bone relationship despite intra-operative micro-motions; (ii) by simplifying setup and avoiding re-registration events, motion-following would shorten operative time; and (iii) by continuously synchronizing the end-effector to bone movement, motion-following would enhance osteotomy fidelity, reflected in smaller resection thickness errors for distal femoral and proximal tibial cuts. Recognizing the centrality of patient-centered outcomes, we retrospectively compared the improvement in validated functional scores at 6 months post-operatively between groups (change from baseline to 6 months), alongside standard radiographic parameters (hip–knee–ankle angle and coronal component angles). By holding the platform, exposure, and implant selection constant, this study isolates the incremental value of motion-following control within RA-TKA and examines its clinical significance through both technical accuracy and functional improvement at 6 months.

## Methods

### Real-time intra-operative motion-following technology

The motion-following control function used in this study was designed to maintain the planned tool–bone relationship during bone resection without rigid limb immobilization. Rather than suppressing limb motion with mechanical fixation, the system continuously tracks intra-operative bone movement and updates the robot arm position in real time to preserve alignment with the planned osteotomy surface.

During surgery, optical tracking markers fixed to the femur and tibia allow a three-dimensional vision camera to monitor knee position continuously. The robotic control system synchronizes the pose of the osteotomy tool with the tracked bone position, dynamically compensating for small intra-operative movements caused by saw vibration, soft-tissue traction, or changes in knee flexion. This real-time tracking enables the robot to maintain the intended cutting trajectory relative to the bone even when the limb is not rigidly fixed.

Compared with conventional robotic workflows that rely on a leg holder to stabilize the limb during cutting, the motion-following approach replaces the traditional “fix-and-cut” paradigm with a real-time tracking-and-compensation strategy. This allows bone resection to be performed without rigid limb fixation while preserving the execution fidelity of the pre-operative surgical plan. A detailed technical description of the coordinate transformations, control algorithms, and system architecture is provided in the Supplementary Material.

A video is provided to demonstrate the real-time motion-following behavior of the robotic system during intra-operative bone cutting (see Supplemental material file).

### Study design and patients

We conducted a single-center retrospective comparative study of 60 patients undergoing primary SkyWalker robot–assisted TKA (MicroPort, Shanghai, China) between September 2022 and August 2024 [18]. Patients were allocated into two cohorts according to the intra-operative technique used: the control group (*n* = 30) underwent RA-TKA without motion-following using a conventional rigid leg holder for limb fixation, and the experimental group (*n* = 30) underwent RA-TKA with the motion-following function enabled and no rigid limb fixation. All cases used the same SkyWalker platform and MicroPort knee implants. Two fellowship-trained orthopaedic surgeons with extensive RA-TKA experience who were already experienced with the SkyWalker robotic system prior to the study period performed all surgeries following standardized workflows.

Inclusion criteria were: idiopathic medial and/or lateral compartment knee osteoarthritis refractory to conservative treatment; age 40–80 years; body mass index (BMI) ≤ 35 kg/m^2^; coronal varus deformity ≤ 15° and fixed flexion contracture ≤ 15°; no prior surgery on the index knee; intact collateral ligaments and overall knee stability; and absence of local or systemic infection. Exclusion criteria were: prior knee arthroplasty on the affected side; flexion contracture or varus deformity > 15°; severe bone/metabolic disease (e.g., tumor, severe osteoporosis) precluding secure component fixation; and inability to understand the procedure or comply with follow-up.

This retrospective study was approved by the medical ethics committee of Shanghai Ninth People’s Hospital, Shanghai Jiao Tong University School of Medicine (SH9H-2025-T521-2).

### Surgical procedure

All procedures were performed under general anesthesia with patients in the supine position. A standard medial parapatellar arthrotomy was used. Pre-operative CT of the limb (hip-to-ankle) was imported into the SkyWalker planning software to generate a 3D model and define target resections (varus/valgus, resection thicknesses, component size) per manufacturer protocol. The robotic platform operates under a semi-active (shared-control) paradigm, providing spatial guidance, virtual boundary protection, and intra-operative calibration while all critical surgical actions remain under surgeon control. The system is an open-implant platform compatible with both posterior-stabilized (PS) and cruciate-retaining (CR) prostheses, and mechanical alignment principles were generally followed. Intra-operatively, bony landmarks were registered using optical trackers, and the robotic arm was positioned to execute the preplanned cuts. The robotic platform supports intra-operative ligament-balance evaluation, including flexion-gap, extension-gap, and dynamic gap assessment.

In the control group, a rigid leg holder immobilized the limb during cutting, and the robot-held guide was aligned under static conditions. In the experimental group, no rigid fixation was applied; the SkyWalker system operated in real-time motion-following mode, continuously tracking limb movement and updating tool pose to maintain the planned cutting trajectory. In both groups, bone cuts were performed with an oscillating saw through robot-held jigs. Trial components were used to confirm ligament balance in flexion/extension; definitive femoral and tibial components were cemented according to plan. Patellar resurfacing and closure followed standard techniques. Apart from limb fixation versus motion-following, all steps (exposure, registration, cutting, trialing, cementation, closure) were identical between groups.

### Outcome measures and data collection

Primary endpoints were surgical efficiency and osteotomy accuracy. Operative time was divided into two components: anesthesia-to-skin time and skin-to-skin time. Anesthesia-to-skin time was defined as the interval from induction of anesthesia to the first skin incision. Skin-to-skin time was defined as the interval from the initial incision to completion of the final skin closure/dressing. All time stamps were extracted from the OR/anesthesia record and rounded to the nearest minute. Leg holder use and intra-operative limb repositioning were both abstracted from the operative note and nursing checklist. Leg holder use was coded as a binary variable (yes/no), where “yes” indicated use of any rigid limb-fixation device during bone preparation. Intra-operative repositioning was defined as any unplanned adjustment of the limb position after registration or during bone-cutting steps.

Osteotomy accuracy was assessed as resection depth error, the absolute difference (millimeters) between planned and achieved resection thickness for each cut. Planned values were taken from the robotic plan. For achieved values, we measured the resected bone thickness intra-operatively with a sterile digital caliper at predefined medial and lateral landmarks, and compared it to the expected thickness calculated from the robotic plan while accounting for cartilage thickness and the cutting-jig gap [19]. Expected thickness = planned resection + measured cartilage − jig gap (1.5 mm); the absolute error was the difference between achieved and expected.

Radiographic alignment: Calibrated, full-length standing anteroposterior radiographs were used to measure the hip–knee–ankle angle (HKA; medial angle between femoral and tibial mechanical axes), the coronal femoral component angle (CFCA; angle between the femoral mechanical axis and the distal tangent of the femoral component), and the coronal tibial component angle (CTCA; angle between the tibial mechanical axis and the proximal tangent of the tibial component). For each parameter, the preplanned target from the robotic plan was recorded, and the absolute deviation from plan was calculated as |Δ| =|post-operative angle − planned angle| (e.g., |ΔHKA| =|HKA_post − HKA_plan|; similarly, for CFCA and CTCA). We report |Δ| (°) as mean ± SD and the proportion ≤ 0.5 [20].

Functional outcome: Patient-reported knee function was assessed using the Western Ontario and McMaster Universities Osteoarthritis Index (WOMAC) collected pre-operatively and at 6 months post-operatively (± 7 days) as part of routine clinical follow-up [21]. We used the Likert version (0–4 per item) and transformed the WOMAC total score to a 0–100 scale, with higher scores indicating better status. The primary functional comparison was the between-group difference in change in WOMAC total score from baseline to 6 months (ΔWOMAC = WOMAC_6-month − WOMAC_pre-op). Absolute WOMAC total scores at 6 months were also compared between groups as a secondary analysis. Patients with both time points available were included in the WOMAC analysis; missing 6-month WOMAC data were not imputed.

Clinical data were extracted from the hospital’s routine database by orthopaedic surgeons not affiliated with the robotic system’s manufacturer. Outcome measurements were performed independently by two orthopaedic surgeons blinded to group allocation, with discrepancies resolved by consensus.

### Radiographic analysis

Long-leg films were obtained with the patella facing forward and standard positioning. Two orthopaedic surgeons, blinded to group allocation, independently measured HKA, CFCA, and CTCA using PACS tools. Each observer performed two readings per film; the mean of the two readings was used for analysis.

### Statistical analysis

Analyses were performed in SPSS v26.0 (IBM, Armonk, NY). Normality of continuous variables was assessed by the Shapiro–Wilk. Continuous variables are reported as mean ± SD (or median [IQR] if non-normal); categorical variables as counts (%). Between-group comparisons used independent-samples t tests for normally distributed variables or Mann–Whitney U tests otherwise; categorical variables used chi-square or Fisher’s exact tests as appropriate.

For WOMAC, the primary comparison was the between-group difference in ΔWOMAC (change from baseline to 6 month). Statistical significance was set at two-sided *p* < 0.05. Given the retrospective design, the sample size reflected available cases; no a priori power calculation was performed.

## Results

### Baseline characteristics

The two cohorts (*n* = 30 each) were broadly comparable at baseline (Table 1). The motion-following group was slightly older (66.5 ± 12.0 vs 63.9 ± 9.1 years) and had a lower mean BMI (27.2 ± 8.1 vs 29.5 ± 5.6 kg/m^2^), whereas the control group included more right knees (18/12). Sex distribution (63.3% vs 53.3% female) and coronal varus deformity (6.9 ± 8.0° vs 5.4 ± 5.1°) were similar. None of these between-group differences reached statistical significance, indicating comparable cohorts (see Table 1 for means ± SD and *p*-values).

**Table 1:** Baseline patient characteristics in each group. Values are mean ± SD or counts. No significant differences between groups (*p* > 0.05 for all comparisons)

Characteristic	Motion-following (*n* = 30)	Control (*n* = 30)	*p*-value
Age, years (mean ± SD)	66.5 ± 12.0	63.9 ± 9.1	0.35
Female sex, *n* (%)	19 (63.3%)	16 (53.3%)	0.43
BMI, kg/m^2^ (mean ± SD)	27.2 ± 8.1	29.5 ± 5.6	0.21
Varus deformity, degrees (mean ± SD)	6.9 ± 8.0	5.4 ± 5.1	0.39
Laterality (left/right), *n*	11/19	18/12	0.07

### Surgical efficiency

Total operative time was significantly shorter in the motion-following group compared with the control group (118.8 ± 9.3 vs. 133.9 ± 11.9 min; *p* < 0.001), representing an approximate 15-min reduction (Table 2).

**Table 2:** Comparison of operative time components between the motion-following and control groups

Operative time	Motion-following (*n* = 30)	Control (*n* = 30)	*p*-value
Total	118.8 ± 9.3	133.9 ± 11.9	< 0.001
Anesthesia to skin	23.4 ± 3.4	30.3 ± 4.0	< 0.001
Skin to skin	95.4 ± 9.4	103.6 ± 11.3	0.003

When the operative time was separated into components, anesthesia-to-skin time was shorter in the motion-following group (23.4 ± 3.4 vs. 30.3 ± 4.0 min; *p* < 0.001), and this phase also showed a complete absence of leg holder use in the motion-following cohort (0% vs. 100%). Skin-to-skin time was likewise reduced (95.4 ± 9.4 vs. 103.6 ± 11.3 min; *p* = 0.003), and intra-operative repositioning occurred in 24 control cases (80%) but in none of the motion-following cases (0%).

### Osteotomy accuracy

Compared with controls, the motion-following cohort showed uniformly smaller resection-thickness errors across all six planes (Table 3). Pooled across planes (equally weighted by plane), the mean error was 0.53 mm in the motion-following group versus 0.82 mm in controls. Aggregating all cuts (6 planes × 30 knees = 180 per group), the proportion ≤ 1 mm was 169/180 (93.9%) with motion-following vs 123/180 (68.3%) in controls; the proportion ≤ 2 mm was 180/180 (100%) vs 175/180 (97.2%).

**Table 3:** Absolute resection-thickness error by cut plane (mm; mean ± SD), with proportions ≤ 1 mm and ≤ 2 mm

Cut plane	Motion-following (*n* = 30)	% ≤ 1 mm	% ≤ 2 mm	control (*n* = 30)	% ≤ 1 mm	% ≤ 2 mm	*p*-value
DF-M	0.48 ± 0.33	96.7%	100%	0.85 ± 0.66	66.7%	96.7%	0.0326
DF-L	0.51 ± 0.33	96.7%	100%	0.86 ± 0.65	63.3%	96.7%	0.0351
PF-M	0.48 ± 0.38	93.3%	100%	0.70 ± 0.42	80.0%	100%	0.0169
PF-L	0.59 ± 0.30	90%	100%	0.69 ± 0.51	76.7%	96.7%	0.7282
TP-M	0.56 ± 0.40	93.3%	100%	0.89 ± 0.66	63.3%	96.7%	0.0933
TP-L	0.53 ± 0.35	93.3%	100%	0.95 ± 0.62	60.0%	96.7%	0.0043

*Abbreviations:* DF-M, Distal femur medial; DF-L, Distal femur lateral; PF-M, Posterior femur medial; PF-L, Posterior femur lateral; TP-M, Tibial plateau medial; TP-L, Tibial plateau lateral

At the plane level, motion-following yielded lower mean errors on every surface, with four planes reaching statistical significance (Table 3). On the distal femur, distal femur medial (DF-M) averaged 0.48 ± 0.33 mm vs 0.85 ± 0.66 mm in controls (*p* = 0.0326; ≤ 1 mm: 29/30 vs 20/30) and distal femur lateral (DF-L) 0.51 ± 0.33 mm vs 0.86 ± 0.65 mm (*p* = 0.0351; ≤ 1 mm: 29/30 vs 19/30). On the tibial plateau, tibial plateau lateral (TP-L) remained significantly improved (0.53 ± 0.35 mm vs 0.95 ± 0.62 mm, *p* = 0.0043; ≤ 1 mm: 28/30 vs 18/30), whereas tibial plateau medial (TP-M) showed the same trend without statistical significance (0.56 ± 0.40 mm vs 0.89 ± 0.66 mm, *p* = 0.0933; ≤ 1 mm: 28/30 vs 19/30). Posterior femoral cuts also followed the same direction, with posterior femur medial (PF-M) showing a significant reduction (0.48 ± 0.38 mm vs 0.70 ± 0.42 mm, *p* = 0.0169; ≤ 1 mm: 28/30 vs 24/30) and posterior femur lateral (PF-L) showing a non-significant difference (0.59 ± 0.30 mm vs 0.69 ± 0.51 mm, *p* = 0.7282; ≤ 1 mm: 27/30 vs 23/30). Overall, motion-following consistently tightened the distribution around the 1-mm benchmark across planes.

### Radiographic alignment

Post-operative coronal alignment closely matched the pre-operative plan in both cohorts (Table 4). Mean absolute deviation from plan (|Δ|) for HKA was 0.92 ± 0.70° in the motion-following group versus 1.22 ± 0.78° in controls (*p* = 0.188). Component deviations were similarly small: |Δ|CFCA 0.76 ± 0.71° vs 1.08 ± 0.77° (*p* = 0.082) and |Δ|CTCA 0.81 ± 0.69° vs 0.94 ± 0.57° (*p* = 0.322). A greater proportion of motion-following knees were within ≤ 1.0° of plan (HKA 66.7% vs 36.7%; CFCA 73.3% vs 50.0%; CTCA 70.0% vs 46.7%). Overall, both techniques achieved alignment within approximately 1° of plan, with a trend toward tighter clustering in the motion-following cohort.

**Table 4:** Postoperative coronal alignment—absolute deviation from plan (|Δ|, °) and ≤ 1.0° proportion: motion-following vs control

Parameter	Motion-following (*n* = 30)	≤ 1.0° (*n*, %)	Control (*n* = 30)	≤ 1.0° (*n*, %)	*p*-value
HKA (°)	0.92 ± 0.70	20/30 (66.7%)	1.22 ± 0.78	11/30 (36.7%)	0.188
CFCA (°)	0.76 ± 0.71	22/30 (73.3%)	1.08 ± 0.77	15/30 (50.0%)	0.082
CTCA (°)	0.81 ± 0.69	21/30 (70.0%)	0.94 ± 0.57	14/30 (46.7%)	0.322

*Abbreviations:* HKA, hip-knee-ankle angle; CFCA, coronal femoral component angle; CTCA, coronal tibial component angle

### Functional outcome

WOMAC total scores improved significantly from baseline to 6 months in both cohorts (within-group *p* < 0.001). Baseline values were comparable (motion-following 40.8 ± 11.8 vs control 38.2 ± 20.5; *p* = 0.55). At 6 months, mean WOMAC increased to 73.6 ± 12.7 in the motion-following group and 68.3 ± 19.4 in controls (*p* = 0.22). The corresponding change (ΔWOMAC = 6-month − pre-op) was 32.8 ± 8.5 versus 30.1 ± 7.9 (*p* = 0.21), indicating substantial functional gains in both groups without a statistically significant between-group difference at 6 months (Table 5).

**Table 5:** WOMAC functional scores before and 6 months after TKA (mean ± SD)

Group (*n* = 30)	Pre-op WOMAC	6-month WOMAC	ΔWOMAC
Motion-following	40.8 ± 11.8	73.6 ± 12.7	32.8 ± 8.5
Control	38.2 ± 20.5	68.3 ± 19.4	30.1 ± 7.9
Between-group *p*-value	0.55	0.22	0.21

*Abbreviations:* WOMAC, Western Ontario and McMaster Universities Osteoarthritis Index

ΔWOMAC = 6-mo − Pre-op

## Discussion

This study found that a real-time, motion-compensating robotic system can streamline the surgical workflow in TKA without sacrificing—or even modestly improving—osteotomy accuracy. By eliminating the need for a rigid leg holder, the motion-following system simplified pre-incision preparation and contributed to a shorter anesthesia-to-skin interval. During the surgical phase, the motion-following system reduced intra-operative repositioning, which was reflected in a shorter skin-to-skin time. Together, these improvements yielded an overall reduction of approximately 15 min in total operative time in our series. Prior studies have identified operative duration as a modifiable risk factor in total joint arthroplasty, with longer procedures associated with increased infection risk [14, 22]. These workflow improvements are consistent with the intended role of motion-following control in maintaining the planned tool–bone relationship during intra-operative micro-motion, thereby reducing interruptions related to limb repositioning or fixation adjustment.

Prior comparisons of robotic-assisted TKA and manual TKA have shown that robotic systems need not prolong surgery time in the hands of experienced teams. Any early inefficiency tends to normalize after a brief learning curve of approximately 9–27 cases (median 17), and with routine use and standardized workflows, robotic TKA can achieve throughput on par with traditional techniques [23]. In short, our findings of improved efficiency align with the literature, noting that dedicated robotic workflows, case clustering, and experienced staff can significantly enhance intra-operative flow.

Importantly, abandoning rigid fixation did not impair accuracy. On the contrary, when the robot continuously adjusted for minor limb shifts, the deviations from the surgical plan were slightly smaller in our motion-following group. This supports the notion that dynamic registration or tracking can obviate mechanical immobilization without loss of precision. A multi-center series of robotic TKA reports that implant positioning and limb alignment accuracy are excellent from the very first case and remain consistent thereafter [3]. Kayani et al. found no “learning curve” effect on accuracy measures: implant orientation and coronal limb alignment were reliably achieved with the robot despite initial longer operative times [24]. Our data extend these findings by showing that even without a conventional leg holder, the robotic system-maintained alignment within planned tolerances. In practical terms, the small gains in precision we observed could translate to fewer recuts and reduced risk of malalignment. Prior reports note that robotic assistance tends to yield fewer alignment outliers than manual techniques, particularly in challenging deformities [25].

This observation is consistent with the motion-following control architecture used in the present study, in which both the patient bone and the robot end-effector are tracked intra-operatively to preserve their relative spatial relationship during osteotomy execution. Compared with conventional navigation-dependent robotic tracking workflows that rely primarily on limb stabilization to maintain execution accuracy, the motion-following system was able to compensate for small intra-operative bone displacements in real time. This capability was supported by the use of a lightweight surgical robotic arm and a high-frequency control architecture, which enabled rapid and stable compensation during bone cutting.

Clinically, these improvements suggest a more surgeon-friendly environment. The workflow in robotic TKA already includes safeguards (such as automatic deactivation with sudden leg movement) that protect against accidental cutting beyond the plan. By actively compensating for minor shifts instead of waiting for error-triggered shutoffs, motion-following can reduce interruptions. Surgeons in our study experienced fewer pauses to reposition the limb or re-secure trackers. This smoother experience—complemented by well-defined checklists and roles in the operating team—may help reduce staff stress and build confidence over time [26]. In the end, both the motion-following and rigid-fixation groups achieved excellent global alignment and component positioning. This aligns with broader evidence that robotic planning and intra-operative guidance help surgeons achieve the intended limb alignment targets consistently [27]. In other words, motion-following seems to refine the execution of a good plan without compromising the overall surgical goals. In addition, the motion-following technology evaluated in this study was implemented on a robotic platform capable of supporting multiple prosthesis systems, allowing the same motion-following control strategy to be applied across different implant designs, including MicroPort, Johnson & Johnson, and Zimmer knee prostheses.

Several limitations of our study should be noted. Its retrospective, single-center design and relatively modest sample size limit broad generalizability. We only evaluated one robotic platform, and results could differ with other systems or in less-experienced settings. We did not measure patient-reported outcomes or longer-term implant survivorship, so we cannot yet say whether the added precision and efficiency will translate into better function or longevity. These limitations mirror those cited by others, who stress the need for prospective, multicenter trials comparing clinical outcomes and cost-effectiveness.

Future work should address these points. A prospective study could validate our efficiency and accuracy findings and assess whether motion-following offers particular advantages in difficult cases. For example, extreme knee deformities or severely osteoporotic bone (where cutting precision and bone support are critical) might benefit most from active tracking. Likewise, measuring patients’ functional scores and quality of life will clarify the clinical impact of this technology. Finally, the core idea of dynamic compensation may apply to other orthopaedic procedures (such as revision joint reconstruction or fracture fixation) where precise alignment is needed without cumbersome jigs.

## Supplementary Information

Supplementary Material 1.

## Data Availability

Data supporting the findings of this study can be obtained from the corresponding author upon reasonable request.
