# Supplementary material for: Real-time intraoperative motion-following robotic assistance improves efficiency and accuracy in total knee arthroplasty: a retrospective comparative study
Source: Arthroplasty. 2026 Apr 14;8:29. doi: 10.1186/s42836-026-00384-9 (PMC13077868; doi:10.1186/s42836-026-00384-9)
Supplement: Supplementary file 1 — Supplementary Material 1. [file 42836_2026_384_MOESM1_ESM.docx]

**Supplemental Material**

**Supplementary 1:**

**Method 1.** Architecture of Real-Time Intraoperative Motion-Following Technology

**Supplementary 2:**

**Fig. S1.** Joint robot leg motion tracking coordinate system

**Fig. S2.** The Motion-Following control scheme of the joint robot system

**Supplementary 3**

**Video 1**. Intraoperative Demonstration of Real-Time Motion-Following During Robotic TKA

**Supplementary 1**

**Method 1.** **Architecture of Real-Time Intraoperative Motion-Following Technology**

The Intraoperative Motion-Following scheme of the robot arm control system designed in this paper is shown as follows (Supplementary Fig. S1):

Robot system coordinate system:

$S_{\text{Robot}}$: represents the joint robot base coordinate system

$S_{\text{RobotTip}}$: represents the coordinate system at the end-tip of the joint robotic arm

$S_{\text{ToolTip}}$: represents the coordinate system of the osteotomy tool on the joint robot

$T_{\text{RobotTip}}^{\text{Robot}}$: represents the spatial transformation from the robot base to the robot arm end-tip

$T_{\text{ToolTip}}^{\text{RobotTip}}$: represents the spatial transformation from the robot arm end-tip to the osteotomy tool

$S_{\text{ToolRef}}$: represents the vision tracking coordinate system of the robot arm.

$T_{\text{ToolRef}}^{\text{NDI}}$: represents the spatial transformation from the NDI camera to the robot arm vision tracking device.

Patient knee joint coordinate system:

$S_{\text{CT\_Volume}}$: represents the coordinate system of the patient’s knee joint preoperative CT image.

$S_{\text{BoneRef}}$: represents the coordinate system of the knee joint vision tracking device (fixed on the femur and tibia)

$T_{\text{CT\_Volume}}^{\text{BoneRef}}$: represents the spatial transformation from the knee joint vision tracking device to the preoperative CT image. It can be calculated and confirmed through Intraoperative bone registration.

$T_{\text{BoneRef}}^{\text{NDI}}$: represents the spatial transformation from the NDI camera to the knee joint vision tracking device.

$S_{\text{Implant}}$: represents the coordinate system of the surgical planning position for the prosthesis implant.

$T_{\text{Implant}}^{\text{CT\_Volume}}$: represents the spatial transformation from the preoperative CT image to the surgical planning position for the prosthesis implant. It can be calculated and confirmed through preoperative surgical planning.

$S_{\text{CutPlane}}$: represents the coordinate system of the planning osteotomy surface.

$T_{\text{CutPlane}}^{\text{Implant}}$: represents the spatial transformation from the surgical planning position of the prosthesis implant to the planning osteotomy surface. It can be calculated and confirmed through parameter configuration for prosthesis osteotomy.

During the intraoperative Motion-Following process, the spatial transformation from the NDI camera to the planning osteotomy surface is shown as follows:

$T_{\text{CutPlane}}^{\text{NDI}}=T_{\text{CutPlane}}^{\text{Implant}}*T_{\text{Implant}}^{\text{CT\_Volume}}*T_{\text{CT\_Volume}}^{\text{BoneRef}}*T_{\text{BoneRef}}^{\text{NDI}}$ (1)

During the Intraoperative Motion-Following process, the coordinate system of the osteotomy tool $S_{\text{ToolTip}}$ would coincide with the coordinate system of the planning osteotomy surface $S_{\text{CutPlane}}$. Namely, the planning osteotomy surface is the target position of the robot osteotomy tool during the Intraoperative Motion-Following control process.

Based on the above intraoperative Motion-Following design, the spatial transformation from the robot base to the planning osteotomy surface would be calculated, and it is also the target control position for the robot arm osteotomy tool during the intraoperative Motion-Following process.

$T_{\text{CutPlane}}^{\text{Robot}}=T_{\text{CutPlane}}^{\text{NDI}}*\left( T_{\text{ToolRef}}^{\text{NDI}} \right)^{-1}*T_{\text{ToolRef}}^{\text{ToolTip}}*T_{\text{ToolTip}}^{\text{RobotTip}}*T_{\text{RobotTip}}^{\text{Robot}}$ (2)

When the patient’s leg moves during the osteotomy process, with the Intraoperative Motion-Following function, the robot arm could track and compensate for the patient's micro-movements in real time during the osteotomy procedure. The Motion-Following control scheme of the joint robot system is shown in Supplementary Fig. S2.

When the patient knee joint moves during the osteotomy process, the 3D vision camera would track the real-time position of the knee joint(femur and tibia), as well as the position of the robot arm, and then calculate the robot volumetric target position for bone osteotomy with the Motion-Following control algorithm, as shown in Algorithm Formula (1) and (2), and then the volumetric target position is sent to the robot arm control unit.

For the robot arm control unit, the robot’s volumetric actual position is calculated with robot’s forward kinematic algorithm, and then the deviation with the volumetric target position is calculated. Thereafter, based on the robot Jacobian matrix, take the inverse to obtain the joint command position deviation ∆*θ_i_*.

For the robot arm control unit, the joint command position (*θ*_cmd_*__i_* =*θ*_cur_*__i_* +∆θ*_i_*) is calculated for each robot joint, and then sent to the joint control mode for robot arm motion control, and execute the Motion-following command.

**Supplementary 2**


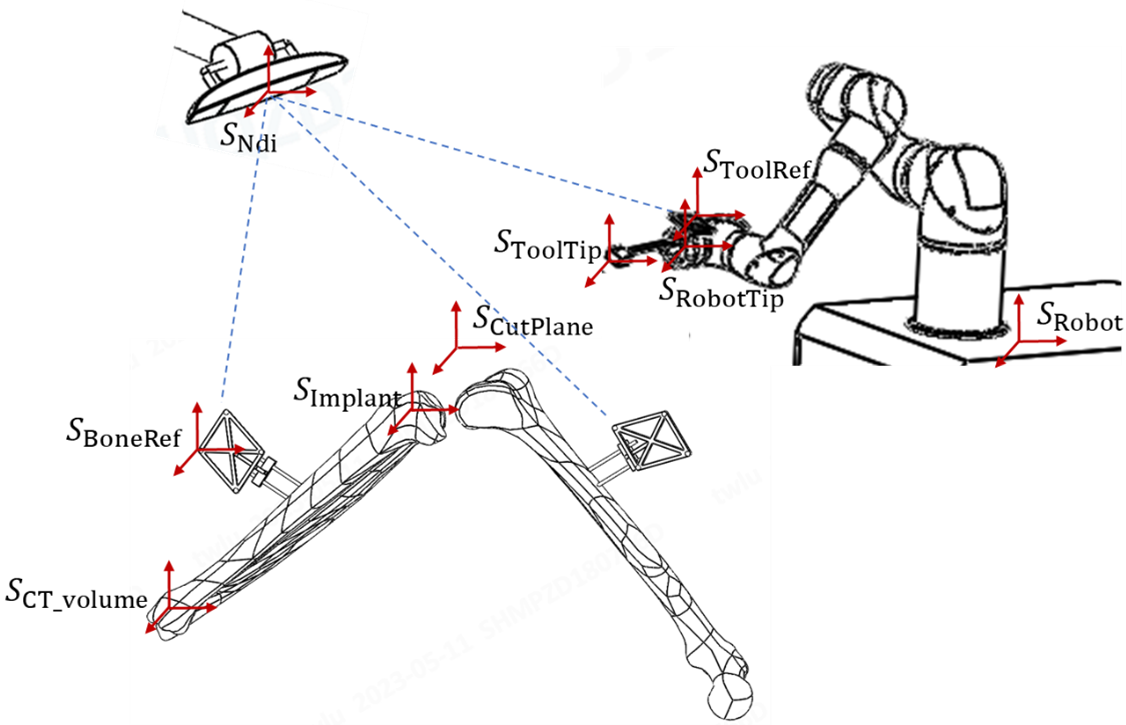


**Fig. S1.** Joint robot leg motion tracking coordinate system


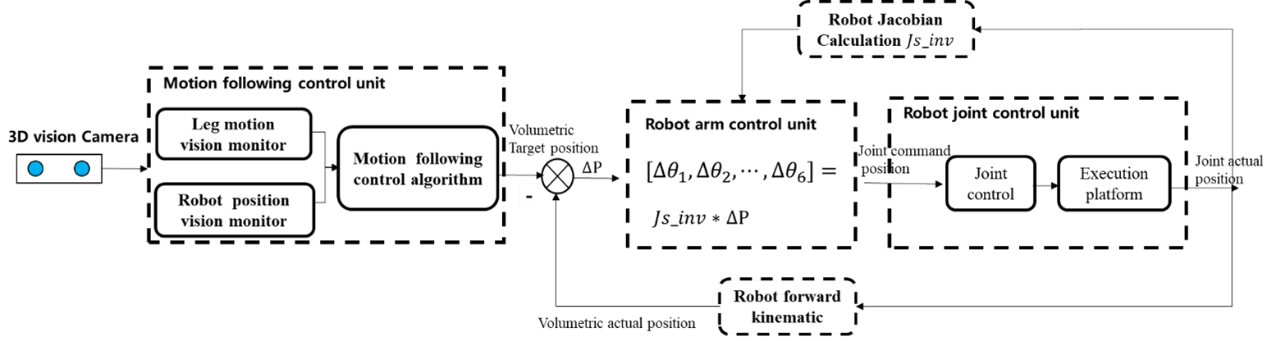


**Fig. S2.** The Motion-Following control scheme of the joint robot system

**Supplementary 3**

**Video 1.** Intraoperative Demonstration of Real-Time Motion-Following During Robotic TKA

**Note:** This video demonstrates the real-time motion-following behavior of the robotic system during intraoperative osteotomy. The system automatically tracks limb movement and dynamically compensates for micro-motion to maintain alignment with the planned resection plane throughout the cutting process.
